# 297. Comparative In Vitro Microbiologic Activity of Two Antimicrobial Intravenous Connectors

**DOI:** 10.1093/ofid/ofae631.087

**Published:** 2025-01-29

**Authors:** Y Lan Truong, Joel Rosenblatt, Bahgat Z Gerges, Ying Jiang, Issam I Raad

**Affiliations:** UT MD Anderson Cancer Center, Houston, Texas; MD Anderson UT, Houston, Texas; MD Anderson UT, Houston, Texas; The University of Texas MD Anderson Cancer Center, Houston, Texas; MD Anderson UT, Houston, Texas

## Abstract

**Background:**

Intravenous (IV) connectors are ubiquitous access junctions in vascular access circuits. In accessing IV connectors (IVCs) for infusions, they become vulnerable to microbial contamination and colonization. Antimicrobial IVCs are treated with antimicrobial agents to resist microbial colonization. In this in vitro study, we compared the microbiologic efficacy of IVCs treated with silver and with a novel coating of Minocycline (M), Rifampin (R) and Chlorhexidine (C).

MRC vs Silver Antimicrobial IV Connectors: 7-Day Microbiologic Challenge Test

**Figure:**
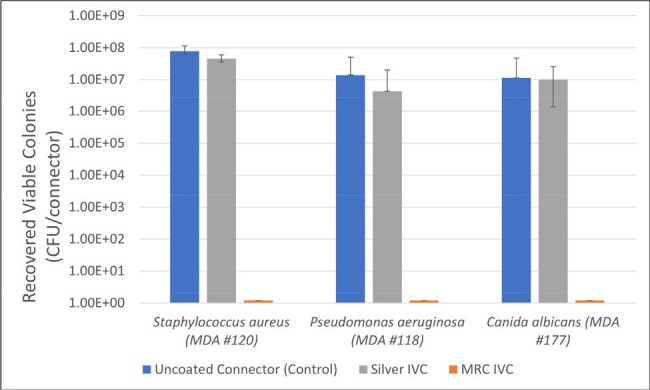

Colonization of MRSA, P. aeruginosa, and C. albicans on silver and MRC IVCs following 7 days of elution. Non-antimicrobial IVCs were used as positive controls. Data is presented as the median CFU/connector from six replicates; bars indicate the range.

**Methods:**

M, R, and C were spray coated onto IVCs in sequential laminate layers to produce MRC IVCs. Commercially available silver IVCs were purchased (Antimicrobial Microclave Connector, ICU Medical Inc., San Clemente, CA). MRC and silver IVCs were immersed separately in tubes filled with 50% serum + 50% saline to simulate elution of the coatings. After 7 days elution (typical interval of use prior to replacement of IV connectors), eluted connectors were exposed to challenge inocula (5x10^5^ CFU/mL) of key CLABSI clinical isolates of methicillin-resistant *Staphylococcus aureus* (MRSA), multidrug-resistant *Pseudomonas aeruginosa* (*PA*), and *Candida albicans* (*CA*) for 24 hours at 37C. The number of viable colonizing microbes (CFU/connector) were enumerated by sonication, serial dilution, plating, and counting. Non-coated IVCs were positive controls.

Log10 Reduction of MRC and Silver IV Connectors vs Positive Control

**Figure:**
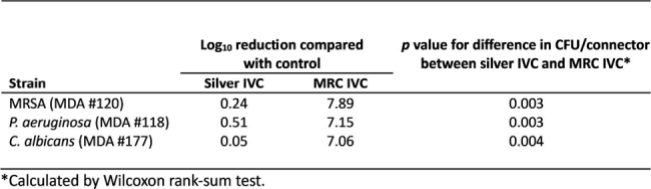

Log10 reduction of the median number of CFU/connector recovered from six replicates following colonization of MRSA, P. aeruginosa, and C. albicans to silver IVCs and MRC IVCs.

**Results:**

Figure 1 presents the median number of CFU/connector with range bars indicating the highest and lowest number of viable colonies recovered from six replicates after 7 days of elution and following 24-hour microbial challenges with MRSA, *PA*, and *CA*. Table 1 shows the median log reductions between positive control and coated connectors with *p*-values comparing MRC and silver IVCs calculated by Wilcoxon rank sum test.

**Conclusion:**

The absence of microbial colonization of the MRC IVCs demonstrates the efficacy and durability of the novel MRC coating. The silver IVC was unable to fully eradicate biofilms of any of the tested pathogens. MRC IVCs were superior to silver IVCs (*p*< 0.05) for all pathogens tested. MRC-coated IVCs merit further testing in vitro and in vivo for their potential to prevent CLABSI.

**Disclosures:**

**All Authors**: No reported disclosures

